# Sensors and Sensor’s Fusion in Autonomous Vehicles

**DOI:** 10.3390/s21196586

**Published:** 2021-10-01

**Authors:** Andrzej Stateczny, Marta Wlodarczyk-Sielicka, Pawel Burdziakowski

**Affiliations:** 1 Department of Geodesy, Gdansk University of Technology, 80-233 Gdansk, Poland; pawel.burdziakowski@pg.edu.pl; 2 Department of Geoinformatics, Maritime University of Szczecin, 70-500 Szczecin, Poland; m.wlodarczyk@am.szczecin.pl

## 1. Introduction

Autonomous vehicle navigation has been at the center of several major developments, both in civilian and defense applications. New technologies such as multisensory data fusion, big data processing, and deep learning are changing the quality of areas of applications, improving the sensors and systems used. New ideas such as 3D radar, 3D sonar, LiDAR, and others are based on autonomous vehicle revolutionary development.

The Special Issue entitled “Sensors and Sensor’s Fusion in Autonomous Vehicles” was focused on many aspects of autonomous vehicle sensors and their fusion, such as autonomous navigation, multi-sensor fusion, big data processing for autonomous vehicle navigation, sensors related to science/research, algorithms/technical development, analysis tools, synergy with sensors in navigation, and artificial intelligence methods for autonomous vehicle navigation. 

Topics for the Special Issue included the following:

- Sensor fusion;
- Sensors based on autonomous navigation;
- Comparative (terrain reference) navigation;
- 3D radar and 3D sonar;
- Gravity and geomagnetic sensors;
- LiDAR;
- Artificial intelligence in autonomous vehicles;
- Big data processing;
- Close-range photogrammetry and computer vision methods;
- Deep learning algorithms;
- Fusion of spatial data;
- Processing of sensors data.

The Special Issue “Sensors and Sensor’s Fusion in Autonomous Vehicles” highlighted a variety of topics related to sensors and sensor’s fusion in autonomous vehicles. The sequence of articles included in this Special Issue is in line with the latest scientific trends. The latest developments in science, including artificial intelligence, were used. The 17 papers (from 28 submitted) was published.

In this article, we provide a brief overview of the published papers, in particular the use of advanced modern technologies and data fusion techniques. These two areas seem to be going in the right direction for the future development of autonomous vehicles navigation.

## 2. Overview of Contributions

As autonomous vehicles have been developing very intensively, recently Arshad S. et al. [1] presented Clothoid—a unified framework for fully autonomous vehicles. In the literature, there are many solutions for autonomous driving frameworks. However, it should be emphasized that building a fully safe and functional system is still a challenge. Even large companies that specialize in building autonomous vehicles unfortunately still cannot avoid accidents. Such examples include the Tesla and Volvo XC90, where serious injuries and even deaths have occurred. This is a consequence of rapid urbanization and the demand for mobility. Nowadays, an autonomous vehicle is expected to have an impact on increasing road safety and thus reduce accidents. All vehicle manufacturers strive to achieve the highest level of autonomy. To achieve this, it is necessary to ensure the accurate detection of the environment and safe driving in different scenarios. The authors proposed a new unified framework for fully autonomous vehicles that integrates multiple modules. Clothoid was implemented on Hyundai I-30 vehicle with a customized sensory and control system. The modules used are described in detail in the system architecture. The proposed solution includes modules that take into account safety, i.e., HD mapping, localization, environment perception, path planning, and control modules. Additionally, comfort and scalability in a real traffic environment were considered. The presented framework enables obstacle avoidance, pedestrian safety of road users, detection of objects, and the avoidance of roadblocks and path planning for single- and multi-lane routes. The authors presented a solution that would allow autonomous vehicles to drive safely throughout the journey. During the tests, the performance of each module was verified and validated in K-City in multiple different situations. During these tests, the proposed Clothoid was safely driven from the starting point to the target point. Note that the proposed vehicle was one of the top five to successfully complete the Hyundai AVC (autonomous vehicle challenge). The proposed framework is enabled to handle the challenging conditions in real environments, including urban areas and highway. Clothoid’s distinguishing characteristics include the ability to deal with difficult situations. These include, for example, detecting and avoiding people on foot, avoiding construction sites and other obstacles, giving way to ambulances or other services, and locating a vehicle in regions where GPS does not work.

Borkowski P. et al., in the work [2], proposed a method of interpolation of the ship’s state vector based on the data from measurements conducted during the sea trials of the ship for determining the anticollision maneuver trajectory. When planning a collision avoidance maneuver in open waters, the ship’s maneuverability and hydrometeorological conditions were taken into account. The ship’s state vector is predicted based on position coordinates, speed, heading, and other movement parameters—at fixed time intervals for different steering scenarios. The proposed function interpolates the parameters of the ship’s state vector for the specified point of a plane, where the values in the interpolation nodes are data obtained from measurements performed during a series of turning circle tests, conducted for different starting conditions and various rudder settings. The mechanism is based on the principles of a modified Dijkstra algorithm, in which the graph takes the form of a regular network of points. The transition between the graph vertices depends on the safe passing level of other objects and the degree of departure from the planned route. The determined shortest path between the starting vertex and the target vertex is the optimal solution for the discrete space of solutions. The presented algorithm was implemented in autonomous sea-going vessel technology. The article presents the results of laboratory tests and tests conducted under quasi-real conditions using physical ship models. The experiments confirmed the effective operation of the developed algorithm of the determination of the anti-collision maneuver trajectory in the technological framework of autonomous ship navigation.

Burdziakowski P. et al. [3] presented a unique combination of bathymetric data obtained from an unmanned surface vessel, photogrammetric data obtained from unmanned aerial vehicles and ground laser scanning, and geodetic data from precision measurements, with receivers of global satellite navigation systems. The article comprehensively describes photogrammetric measurements made from unmanned aerial vehicles during measurement campaigns. Several measurement campaigns took place in the littoral zone in Sopot, related to the intensive uplift of the seabed and beach caused by the tombolo phenomenon. These phenomena cause continuous and multidimensional changes in the shape of the seabed and the Earth’s surface, and when they occur in an area of intense human activity, they should be constantly monitored. The article describes in detail the problems in reconstruction within the water areas, analyzes the accuracy of various photogrammetric measurement techniques, proposes a statistical method of data filtration, and presents the changes that occurred within the studies area. The work ends with an interpretation of the causes of changes in the land part of the littoral zone and a summary of the obtained results. 

In their work [4], Chang L. et al. proposed a multi-sensor integrated navigation system composed of global navigation satellite system (GNSS) inertial measurement unit (IMU), odometer (ODO), and light detection and ranging simultaneous localization and mapping (LiDAR-SLAM). The dead reckoning results were obtained using IMU/ODO in the front end. The graph optimization was used to fuse the GNSS position, IMU/ODO pre-integration results, and the relative position and relative attitude from LiDAR-SLAM to obtain the final navigation results in the back end. The odometer information is introduced in the pre-integration algorithm to mitigate the large drift rate of the IMU. The sliding window method was also adopted to avoid the increasing parameter numbers of the graph optimization. Moreover, land vehicle tests were conducted in both open-sky areas and tunnel cases. As the tests showed, the proposed navigation system can effectually improve the accuracy and robustness of navigation. During the navigation drift evaluation of the mimic two-minute GNSS outages, compared to the conventional GNSS/INS (inertial navigation system)/ODO integration, the root mean square (RMS) roots of the maximum position drift errors during outages in the proposed navigation system were reduced by 62.8%, 72.3%, and 52.1%, along the north, east, and height, respectively. What is more, the yaw error was reduced by 62.1%. If we compare it to the GNSS/IMU/LiDAR-SLAM integration navigation system, the assistance of the odometer and non-holonomic constraint reduced the vertical error by 72.3%. The test conducted in the real tunnel case showed that in weak environmental feature areas where the LiDAR-SLAM barely works, the assistance of the odometer in the pre-integration is critical and can effectively reduce the positioning drift along the forward direction and maintain the SLAM in the short-term. Therefore, the proposed GNSS/IMU/ODO/LiDAR-SLAM integrated navigation system can effectively fuse the information from multiple sources to maintain the SLAM process and significantly mitigate navigation error, especially in harsh areas where the GNSS signal is severely degraded and environmental features are insufficient for LiDAR-SLAM.

In their publication, Chen B. et al. [5] proposed a new algorithm framework of target level fusion of a millimeter-wave radar and a camera. Intelligent autonomous vehicles while driving should detect the target very accurately. This is the basis of safe driving. It is very common that the sensors used today to detect targets have some defects at the perceptual level. The mentioned defects can be compensated by sensor fusion technology, as proposed in this paper. In this research, the authors adopted a certain fusion hierarchy at the target level. The fusion algorithm was divided into two tracking processing modules (for a millimeter-wave radar and a camera) and one fusion center module based on the distributed structure. The measurement information output by the sensors enters the tracking processing module. Then, after processing by a multi-target tracking algorithm, the local tracks are generated and transmitted to the fusion center module. The authors have described these processes in detail and illustrated them with figures. In the fusion center module, a two-level association framework is designed based on local collision association and weighted track association. The association between the sensors’ regional tracks is completed, and a non-reset federated filter is used to calculate the state of the fusion tracks. In the experiments, the camera was installed in the windshield inside the longitudinal symmetry plane of the ego vehicle on the side of the cab, and the radar was installed in the middle of the front bumper of the ego vehicle. The experiment was carried out in an urban road environment, for example, streets, expressways, tunnels, etc. The authors chose single-target fusion, multi-target fusion, and sensor fusion application in detecting dangerous targets. In all experiments, the association for different local traces of the same target is good. The overall performance of trace state estimation is better than that of a single sensor. In the experiment of selecting dangerous targets, the fusion algorithm can replace the dangerous targets more accurately and timely. The publication shows that the proposed algorithm can complete a tracks association between the millimeter-wave radar and camera. The fusion track state estimation method has extremely good performance and can be applied in practice.

Another publication that refers to navigation system is [6]. Feriol F. et al. presented the review of existing GNSS and on-board vision-based solutions of environmental context detection. The current autonomous vehicle navigation systems use data acquired from multiple sensors. This is important to improve their location accuracy, but it is also important to note that there is often uncertainty about the quality of these measurements and the accuracy of these data. The situation in which these data are collected and analyzed is also of great importance. The authors based their study on the statement that the context detection would enable one to create an adaptive navigation system to increase the accuracy and the robustness of its localization solution by anticipating possible degradation in sensor signal quality. The authors consider that the problem of context detection is the future of navigation systems, but the current environmental context detection methods are not robust, since using a single-dimension descriptor is not enough. There is no such solution in the literature trying to combine vision and GNSS indicators. Most of the state-of-the art research articles focus on only one type of data. The future work of authors will include the development of a new algorithm to detect the environmental context consistently based primarily on vision but with the aid of GNSS indicators for navigation adaptation purpose.

Autonomous vehicles are on the rise and are expected to be on the roads in the coming years. In this sense, it is necessary to have adequate knowledge about their states to design controllers capable of providing adequate performance in all driving scenarios. Sideslip and roll angles are critical parameters in vehicular lateral stability. The latter has a high impact on vehicles with an elevated center of gravity, such as trucks, buses, and industrial vehicles, among others, as they are prone to rollover. Due to the high cost of the current sensors used to measure these angles directly, González, L.P. et al., in their work [7], proposed an inexpensive but powerful model based on deep learning to estimate the roll and sideslip angles simultaneously in mass production vehicles. The model uses input signals which can be obtained directly from onboard vehicle sensors, such as longitudinal and lateral accelerations, steering angle and roll and yaw rates. The model was trained using hundreds of thousands of data provided by Trucksim^®^ and validated using data captured from real driving maneuvers using a calibrated ground truth device such as VBOX3i dual-antenna GPS from Racelogic^®^. The use of both Trucksim^®^ software and the VBOX measuring equipment is recognized and widely used in the automotive sector, providing robust data for the research shown in the article.

In order to reduce the cost of the flight controller and improve the control accuracy of the solar-powered unmanned aerial vehicle (UAV), Guo A. et al. in the article [8] proposed three state estimation algorithms based on the extended Kalman filter (EKF) with different structures: three-stage series and full-state direct and indirect state estimation algorithms. A small hand-launched solar-powered UAV without ailerons was used as the object with which to compare the algorithm structure, estimation accuracy, and platform requirements and application. The three-stage estimation algorithm has a position accuracy of 6 m and is suitable for low-cost, small, low-control, and precision UAVs. The precision of full-state direct algorithm is 3.4 m, which is suitable for platforms with low-cost and high-trajectory tracking accuracy. The precision of the full-state indirect method is similar to the direct, but it is more stable for state switching and overall parameters estimation and can be applied to large platforms. A full-scale electric hand-launched UAV loaded with the three-stage series algorithm was used for the field test. Results verified the feasibility of the estimation algorithm, and it obtained a position estimation accuracy of 23 m.

Autonomous surface vehicles with optical systems can be used during shoreline detection and land segmentation [9]. Hozyn and Zalewski believe that optical systems can interpret the surrounding landscape. This is important for navigation through restricted areas and requires advanced and modified image segmentation algorithms. The authors, in their research, analyzed the traditional methods of image processing and neural networks. The solutions based on traditional methods require a set of parameters before execution, and the other, neural-network-based solutions require a very large database. To avoid these problems, the authors used adaptive filtering and progressive segmentation. The first is used to suppress the weak edges of the image, which is very useful during shoreline detection. On the other hand, the progressive segmentation process is mainly aimed at distinguishing between sky and land areas. This method uses a probabilistic clustering model to improve the performance, which gives very good results. The proposed method consists of four main steps: image pre-processing, edge detection, shoreline detection, and progressive land segmentation. The authors conducted a study on images acquired from an operational camera mounted on an autonomous vehicle. It used 1500 images of the city of Gdynia, which is a port city located in Poland. The images show coastal areas (with the shoreline visible) at different distances from land and under different weather conditions. The authors compared the obtained results with existing methods. They show that their method has higher reliability. In most of the tested cases, the developed method correctly performs shoreline detection and land segmentation. The method works regardless of the autonomous vehicle’s distance from the land or the weather conditions in the study area. 

In Kim T. and Park T-H. [10], an extended Kalman filter was proposed to reflect the distance characteristics of LiDAR and radar sensors. The distance characteristics of LiDAR and radar sensors were analyzed, and a reliability function was designed to extend the Kalman filter to reflect the distance characteristics. The accuracy of position estimation was improved by identifying sensor errors as a function of distance. Experiments were conducted on real vehicles, and a comparison experiment combining sensor fusion using a fuzzy filter, an adaptive noise measure, and a Kalman filter was performed. The experimental results showed that the method used provides accurate distance estimation.

In Koszelew J. et al. [11], the problem of anti-collision trajectory planning in multi-vessel encounter situations in the aspect of autonomous navigation of surface vehicles is clarified. The proposed original algorithm (multi-surface vehicle beam search algorithm), based on beam search strategy, solves this problem. The general idea of the proposed algorithm is to apply a solution to a one-to-many encounter situation (using the beam search algorithm), which was tested on real data from a marine navigation radar and automatic identification system. The algorithm’s test results were derived from simulated data, which are discussed in the final section. This paper clarifies the problem of anti-collision trajectory planning in many-to-many encounter situations involving moving autonomous surface vehicles, excluding collision laws and surface vehicle dynamics.

Liu T. et al. [12] proposed a SLAM (simultaneous localization and mapping) scheme using GNSS (global navigation satellite system), IMU (inertial measurement unit) and LiDAR (light detection and ranging) sensor, using the position of pole-like objects as features for SLAM. The scheme combines a traditional preprocessing method and a small-scale artificial neural network to extract pole-like objects in the environment. First, the threshold-based method is used to extract pole-like object candidates from the point cloud, and then, the neural network is used to train and infer pole-like objects. The result shows that the accuracy and recall rate are sufficient to provide stable observation for the following SLAM process. After the poles are extracted from the LiDAR point cloud, their coordinates are added to the feature map, and non-linear front-end optimization is performed by using distance constraints corresponding to the pole coordinates; then, the heading angle and horizontal plane offset are estimated. Terrain feature points are used to improve the accuracy of elevation, pitch, and roll angle measurements. The performance of the proposed navigation system is evaluated through field experiments by checking the position drift and attitude errors during multiple two-minute GNSS mimics without additional IMU motion constraint such as NHC (nonholonomic constrain). Experimental results show that the performance of the proposed system is better than the conventional feature point grid-based SLAM with the same background, especially at busy intersections, where slow moving vehicles are surrounded and pole-like objects are abundant in the environment. The proposed SLAM system based on GNSS/IMU/LiDAR pole-like features can effectively combine the condensed information from these sensors to mitigate positioning and orientation errors, even under short-term GNSS-free conditions.

In the article by Mohammed A.S. et al. [13], an overview of sensor technologies used to detect the environment and obstacles during driving maneuvers in various weather conditions is presented. First, some important historical milestones are presented. Second, state-of-the-art applications for automated driving (adaptive cruise control, pedestrian collision avoidance, etc.) are presented. Third, the most involved sensor technologies (radar, LiDAR, ultrasound, camera, and far infrared) used in automated driving applications are reviewed. Furthermore, the difference between the current and expected performance states is determined using spider graphs. As a result, a fusion perspective is proposed that can bridge the gaps and increase the robustness of the perception system.

Muhovič J. and Perš J. [14] proposed an on-the-fly stereo recalibration method applicable in real-world autonomous vehicles. Due to the fact that camera systems in autonomous vehicles are subject to various sources of anticipated and unanticipated mechanical stress (vibration, rough handling, and collisions) in real-world conditions, even moderate changes in camera geometry decalibrate multi-camera systems and corrupt downstream applications such as depth perception. The presented method is comprised of two parts. First, in the optimization step, external camera parameters are optimized with the goal to maximize the amount of recovered depth pixels. In the second step, an external sensor is used to adjust the scaling of the optimized camera model. The method is lightweight and fast enough to run in parallel with stereo estimation, thus allowing an on-the-fly recalibration. The extensive experimental analysis showed that the method achieves stereo reconstruction better, or on par with, manual calibration. If the method is used on a sequence of images, the quality of calibration can be improved even further.

Another example of data acquired from modern systems and sensors mounted on autonomous vehicles is bathymetry data. These data are depth points acquired from a multi-beam echo sounder. Wlodarczyk-Sielicka and Blaszczak-Bak deal with the problem of their processing [15]. A multi-beam echosounder (MBES collects very large sets of bathymetric points—spatial data with location and depth). The development and analysis of such large datasets are laborious and expensive. In this case, the reduction of such data is a necessary action. In commercial programs, during the reduction of bathymetric data, the methods of interpolation to a specific mesh size are currently widely used. The authors of this publication previously proposed original reduction methods, which maintain the true position and depth for each of the measured points under water. Wlodarczyk-Sielicka developed the true bathymetric data reduction method (TBDRed) that is based on artificial neural networks, whereas Blaszczak-Bak developed a method which is based on Douglas–Peucker generalization. The authors have proposed a fusion of these two methods, with very satisfactory results. It is an innovative approach to the problem of bathymetric data processing and reduction. The authors conducted research on two test basins (inland and offshore) with different characteristics. The results were analyzed visually and statistically in detail. The methods work differently: the TBDRed method allows for a more even distribution of points, and the OptD method allows for a different degree of reduction in areas of the tested object. The resulting post-fusion datasets give a very good representation of the bottom in the study area. The fusion has allowed the maintenance of extreme or important depths and improving the efficiency and speed of generating seabed models. The bottom outcome models in the study areas are fully presented and have the correct shape.

The autonomous emergency braking pedestrian (AEB-P) system has the functional requirements of avoiding the pedestrian collision and ensuring the pedestrian’s life safety. By studying relevant theoretical systems, such as time to collision (TTC) and braking safety distance, an AEB-P warning model and the traffic safety level and work area of the AEB-P warning system were presented by Yang W. et al. in the work [16]. The upper-layer fuzzy neural network controller of the AEB-P system was designed, and the BP (backpropagation) neural network was trained by the collected pedestrian longitudinal anti-collision braking operation data of experienced drivers. Moreover, the fuzzy neural network model was optimized by introducing the genetic algorithm. The lower-layer controller of the AEB-P system was designed based on the proportional integral derivative controller (PID) theory, which realizes the conversion of the expected speed reduction to the pressure of a vehicle braking pipeline. The relevant pedestrian test scenarios were set up based on the C-NCAP (China-new car assessment program) test standards. The CarSim and Simulink co-simulation model of the AEB-P system was established, and a multi-condition simulation analysis was performed. The results showed that the proposed control strategy was credible and reliable and could flexibly allocate early warning and braking time according to the change in actual working conditions, to reduce the occurrence of pedestrian collision accidents.

Very often, autonomous vehicles are equipped with remote sensing technology and wireless multimedia sensor networks. These sensors are able to collect a lot of data, such as video and audio streams, still images, and scalar sensor data from the environment. Zhou B. et al. pointed out that the features extraction and matching are the basis of many high-level applications, including those mounted and used in the navigation of unmanned vehicles [17]. The mentioned article successively shows the related fundamentals of remote sensing monitoring, target tracking, and features extraction, and the multi-level features extraction and discontinuous targets tracking. In the next section, we see several experiments that verify the accuracy and efficiency of the proposed method. The authors proposed a multi-level features extraction for discontinuous target tracking in remote sensing image monitoring. The method is based on the fact that, at first, the features of the reference image are pre-extracted at different levels. Thus, the features on first level are used to roughly check the candidate targets. By contrast, subsequent levels are used for refined matching. The relevant neighborhood can be set adaptively, and a primary parameter analysis is used to improve the descriptor. The authors have clearly demonstrated that their experiments verify the effectiveness and accuracy of their proposed method.

## 3. Conclusions

The Special Issue entitled “Sensors and Sensor Fusion in Autonomous Vehicles” comprised 17 articles on many topics related to navigational aspects of sensors and sensor fusion. In this paper, we have presented short introductions of the published articles.

It can be said that autonomous navigation and sensor’s fusion still remain an important and hot topic, and a lot of work will continue to be conducted worldwide. New techniques and methods for analyzing and extracting information from navigational sensors and their data fusion have been proposed and verified. Some of these will provoke further research, and some are already mature and can be considered for industrial implementation and development.
